# Effect of Chromium on Antioxidant Potential of *Catharanthus roseus* Varieties and Production of Their Anticancer Alkaloids: Vincristine and Vinblastine

**DOI:** 10.1155/2014/934182

**Published:** 2014-03-10

**Authors:** Vartika Rai, Pramod Kumar Tandon, Sayyada Khatoon

**Affiliations:** ^1^Department of Botany, University of Lucknow, Lucknow 226 007, India; ^2^Pharmacognosy & Ethnopharmacology Division, CSIR-National Botanical Research Institute, Lucknow 226 001, India

## Abstract

*Catharanthus roseus* (L.) G. Don, a medicinal plant, has a very important place in the traditional as well as modern pharmaceutical industry. Two common varieties of this plant *rosea* and *alba* are named so because of pink and white coloured flowers, respectively. This plant comprises of about 130 terpenoid indole alkaloids and two of them, vincristine and vinblastine, are common anticancer drugs. The effect of chromium (Cr) on enzymatic and non-enzymatic antioxidant components and on secondary metabolites vincristine and vinblastine was studied under pot culture conditions of both varieties of *C. roseus*. Antioxidant responses of these varieties were analyzed under 0, 10, 50, and 100 **μ**M chromium (Cr) level in order to investigate the plant's protective mechanisms against Cr induced oxidative stress. The results indicated that Cr affects all the studied parameters and decreases growth performance. However, vincristine and vinblastine contents were increased under Cr stress. Results are quite encouraging, as this plant shows good antioxidant potential and increased the level of active constituents under Cr stress.

## 1. Introduction

The pollution is increasing in the environment by different kinds of human activities, which results in the accumulation of heavy metals in the soil and water and it causes different types of problems to the living beings [1]. Heavy metal contaminated lands are increasing in India day by day due to rapid industrialization, urbanization, and mining activities. One cannot use these lands for the agricultural purposes because, in that case, edible crops may accumulate high level of toxic metals which is detrimental for the health of people. In some previous experimental studies [2–5], it was observed that in higher concentration of heavy metals, the percentage yield of secondary metabolites, responsible for therapeutic activities, enhanced in medicinal plants. In this context, one can think on the line of increasing active constituents of some very important medicinal plants by growing them on otherwise wastelands contaminated with heavy metals. Plants, which are used in the modern drug industry and particularly have lower percentage of active constituents, may be selected for such type of study. In industrial crops, such as medicinal plants, the content of the economically important metabolite is more important than the yield of the plant part containing the metabolite, as it determines the cost of its extraction [6].

*Catharanthus roseus* (Linn.) G. Don is a renowned medicinal plant, belonging to the family Apocynaceae, and is a rich source of alkaloids, which are distributed in all parts of the plant. The plant contains about 130 alkaloids of the indole group out of which 25 are dimeric in nature [7]. Two of the dimeric alkaloids, vinblastine and vincristine mainly, present in the aerial parts, have found extensive application in the treatment of human neoplasma [8]. Vinblastine sulphate (sold as Velban) is used particularly to treat Hodgkin's disease besides lymphosarcoma, choriocarcinoma, neuroblastoma, carcinoma of breast and lungs, and other organs in acute and chronic leukemia. Vincristine sulphate (sold as Oncovin) arrests mitosis in metaphase and is very effective for treating acute leukaemia in children and lymphocytic leukemia. It is also used against Hodgkin's disease, Wilkins's tumor, neuroblastoma, and reticulum cell sarcoma [8]. High demand and low yield of these alkaloids in the plant have led to research for alternative means for their production.

Chromium (Cr) is selected as test metal because Cr compounds have various industrial uses and extensively employed in leather processing, production of refractory steel, and specialty chemicals. These anthropogenic activities have led to the widespread contamination of Cr and increased bioavailability and biomobility of Cr. The leather industry is the major cause for the high influx of Cr to the biosphere, accounting for 40% of the total industrial use [9]. In India, about 20000–32000 tons of elemental Cr annually escapes into the environment from tanning industries [10].

Cell and tissue culture and biotechnological aspects of* C. roseus* are being extensively investigated to increase the yield of these alkaloids [11, 12]. Yet, comparatively very little work has been done on the effect of heavy metals on the antioxidant enzymes and alkaloids content of this economically important medicinal plant. Therefore,* C. roseus* has been selected for the present study to know the effect of chromium (Cr) on its antioxidant potential and production of anticancer alkaloids vincristine and vinblastine. Two varieties of this plant, based on the flower colour, namely, pink flowered* rosea* and white flowered* alba*, were selected for the present study.

## 2. Materials and Methods

### 2.1. Sample Preparation

Seeds of the two varieties of* Catharanthus roseus* (L.) G. Don., that is,* rosea* and* alba,* were collected from the botanical garden of Lucknow. The experiments were conducted at the Department of Botany, Lucknow University. The seeds were surface sterilized in a 0.2% HgCl_2_ solution for 5 min (with frequent shaking), and then thoroughly washed with tap water. The seeds were presoaked in 500 mL of water and were sown in specially prepared nursery in the field receiving normal daylight, temperature, and humidity. Plants having approximately the same height and weight were carefully uprooted after 45 days of sowing and transferred into the earthen pots (300 mm diameter) filled with a garden soil (5 Kg) mixed with potassium dichromate (K_2_Cr_2_O_7_) in the concentration of 10, 50, and 100 *μ*M. Three replicates for each concentration were made. The five seedlings approximately of 4 cm height and 1 g fresh weight (FW) were planted in each pot. The plants were watered daily and grown for 30 days and care was taken to avoid leaching of water from the pots. Plants in the soil without Cr treatment served as control. After harvesting, plants were washed under running tap water to remove the soil from root completely and again washed with glass distilled water. The roots and shoots were separated manually, just above the ground level.

### 2.2. Estimation of Chlorophyll

Chlorophyll was estimated by the method of Arnon [13] and absorbance was measured at 663, 645, 510, and 480 nm using Pharmacia Biotec, UV/Vis spectrophotometer.

### 2.3. Estimation of Protein

Protein was estimated by the methods of Lowry et al. [14]. 100 mg plant material was homogenized with 3 mL of 10% trichloroacetic acid. The homogenate was centrifuged at 10,000 rpm and supernatant was discarded. The pellets obtained after centrifugation were treated with 3 mL 1 N NaOH, heated on water bath for 7 minutes and cooled down to room temperature. Again, the solution was centrifuged for five to ten minutes at 5000 rpm. Five mL reagent containing 100 parts of 2% solution of sodium carbonate and one part of 2% solution of sodium potassium tartrate was added to 0.5 mL of supernatant thus obtained after centrifugation and allowed it to stand for ten to fifteen minutes. Then 5 mL Folin and Ciocalteu's Phenol reagent (diluted with distilled water in ratio of 1 : 1) was added and allowed to stand for half an hour for development of colour, and then finally absorbance was measured at 700 nm.

### 2.4. Estimation of Cysteine and Proline Contents

Known amount of plant material was extracted in 5% chilled HClO_4_ and centrifuged at 10000 rpm for 20 min at room temperature. Cysteine content was measured in supernatant using acid ninhydrin reagent by reading absorbance at 560 nm [15]. Proline content was estimated by the method of Bates et al. [16]. The plant material was homogenized in 3% aqueous sulfosalicylic acid. The homogenate was centrifuged at 10000 rpm. Supernatant was used for the estimation of proline content. The reaction mixture consisted of 1.0 mL supernatant, 1.0 mL acid ninhydrin, and 1.0 mL glacial acetic acid, which was boiled at 100°C for 1 hr. After termination of reaction in ice bath, the reaction mixture was extracted with 4 mL of toluene and absorbance was read at 520 nm.

### 2.5. Estimation of Nonprotein Thiol (NP-SH)

For estimation of nonprotein thiol (NP-SH) content, plant tissue was extracted in 6.67% 5-sulfosalicylic acid and centrifuged at 13,000 rpm for 10 min. Supernatant was reacted with Ellman's reagent. Absorbance was recorded at 412 nm [17].

### 2.6. Estimation of Lipid Peroxidation

The level of lipid peroxidation in fresh leaves was measured in terms of malondialdehyde (MDA) content by the thiobarbituric acid (TBA) reaction method [18]. 0.5 g fresh leaves was extracted with 5 mL of 1% TCA, and then centrifuged at 10000 rpm for 5 min. 0.5% TBA in 20% TCA was added in 1 mL of supernatant and heated at 95°C for 30 min, quickly cooled on ice bath, and centrifuged at 10000 rpm for 10 min. Absorbance of the supernatant was taken at 532 nm and 600 nm.

### 2.7. Assay of Antioxidant Enzymes

The samples were prepared as described by Mukherjee and Choudhuri [19]. Plant material was frozen in liquid nitrogen and finely ground by pestle in a chilled motor; the frozen powder was added to 10 mL of 100 mM phosphate buffer (KH_2_PO_4_/K_2_HPO_4_) pH 7.0, containing 0.1 mM Na_2_EDTA and 0.1 g of polyvinylpyrrolidone (PVP). The homogenate was filtered through four layers of cheese cloth and centrifuged at 15000 rpm for 10 min at 4°C. The supernatant obtained will be designated as crude enzyme extract and was used for various antioxidant enzyme assays.

#### 2.7.1. Ascorbate Peroxidase (APX; EC 1.11.1.11)

Ascorbate peroxidase activity was measured in the leaves of the plant by the method of Nakano and Asada [20], estimating the ascorbate oxidation at 290 nm. The activity was calculated using the extinction coefficient of 2.8 mM^−1^ cm^−1^.

#### 2.7.2. Guaiacol Peroxidase (GPX; EC 1.11.1.7)

Guaiacol peroxidase was measured in plant, following the method of Hemeda and Klein [21]. Activity was calculated using the extinction coefficient of 26.6 mM^−1^ cm^−1^ at 470 nm for oxidized tetraguaiacol polymer. One unit of peroxidase activity was defined as the calculated consumption of 1 *μ*mol of H_2_O_2 _min^−1^ g^−1^ fresh weight.

#### 2.7.3. Catalase (CAT, EC 1.11.1.6)

The activity of catalase (CAT) was measured monitoring H_2_O_2_ decomposition at 240 nm in 3 mL reaction mixture containing 50 mM phosphate buffer (pH 7.0), 15 mM H_2_O_2_, 100 *μ*L homogenate, and 0.1% (v/v) Triton X-100 [22]. The activity was expressed in terms of *μ*mol of H_2_O_2_ reduced min^−1^ g^−1^ FW at 25 ± 2°C.

### 2.8. Estimation of Metal Concentration

1 g dried powdered plant material of each treatment was taken and subjected to wet digestion in 3 : 1, v/v, HNO_3_ : HClO_4_. Cr concentration was estimated in Atomic Absorption Spectrophotometer (Perkin-Elmer, A-Analyst 300) [23].

### 2.9. Estimation of Vincristine and Vinblastine through High Performance Thin Layer Chromatography

1 g dried powdered plant material of each treatment was extracted with 20 mL methanol on water bath, consecutively three times. Methanol soluble extracts were filtered and concentrated at low temperature and reduced pressure. 10 mg/mL solution of each extract was prepared with methanol [24]. 1 mg of standards-vinblastine and vincristine sulphate (Sigma) was dissolved in 1 mL of methanol. 20 *μ*L of plant extracts was applied on HPTLC precoated silica gel plates (E. merck 60 F_254_) with the help of CAMAG Linomat 5 applicator. The plates were eluted to the distance of 8.0 cm in the solvent system developed through trial and error basis in previously saturated twin trough chamber (CAMAG). The plates were scanned using CAMAG TLC Scanner 3 with software WinCats 3.2.1. Photographs of TLC plates were taken by the CAMAG video documentation unit Reprostar 3.

### 2.10. Statistical Analysis

All values reported in this work are means of three independent determinations. The mean values ± SD are given in tables and graphs. All the data has been statistically analyzed by one-way analysis of variance (ANOVA) in randomized complete block design to check the variability of data and validity of results. Comparison between control and treatment was done by LSD test [25].

## 3. Results

When* C. roseus* plants were transplanted to soil containing variable concentrations of chromium, at the beginning they showed the symptoms of chlorosis and senescence. But after a period of one week of transfer, the plants gradually adapted and grew well. It seemed that after an initial shock plants grew well. Plants treated with Cr at 10 and 50 *μ*M levels grew normally as shown by leaf numbers, plant height, and biomass. Only at 100 *μ*M level there is some stunted growth (data not shown).

### 3.1. Chromium Accumulation and Its Effect on Chlorophyll and Carotenoids Content

*C. roseus *accumulated chromium in concentration dependent manner (Table 1). The amount of chromium accumulated by different plant tissues (roots > leaves) varied significantly (ANOVA, *P* < 0.05). Roots of* rosea* variety accumulated maximum amount (41.4 ppm DW) of chromium when exposed to 100 *μ*M chromium for 30 days. The maximum chromium (25.96 ppm DW) content in shoots was also recorded at the same concentration and in the same variety.

**Figure 1:**
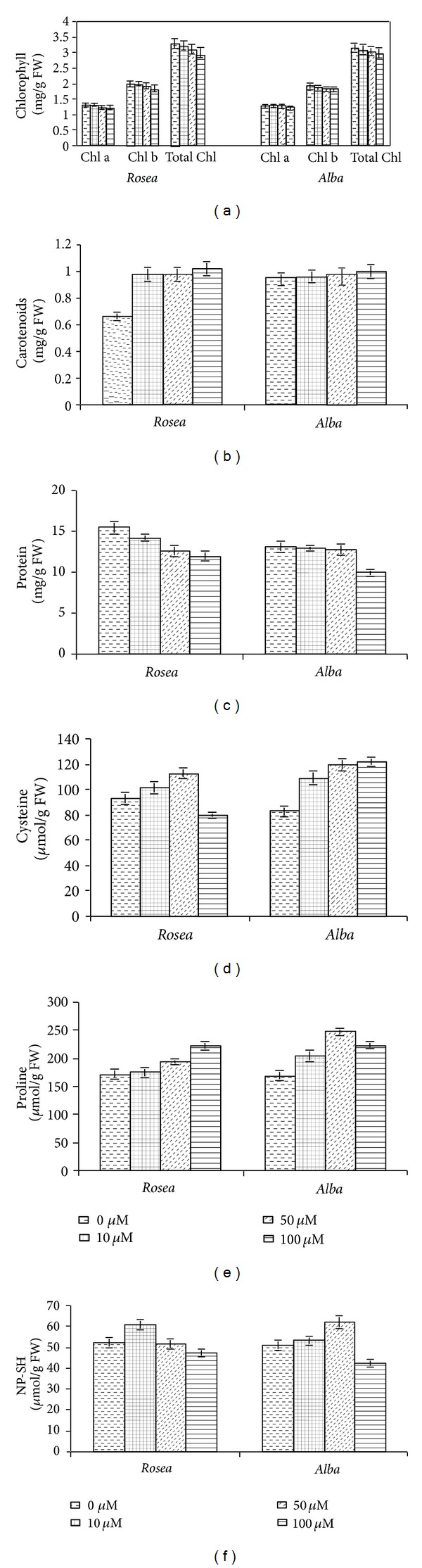
Effect of different chromium concentrations on (a) chlorophyll, (b) carotenoids, (c) protein, (d) cysteine, (e) Proline, and (f) NP-SH content of* C. roseus* after 30 days of Cr treatment. Mean ± S.D. (*n* = 3). ANOVA, *P* < 0.05.

Chromium reduced the level of photosynthetic pigments (total chlorophyll, chlorophyll a, and chlorophyll b) in* C. roseus* (Figure 1(a)). The chromium toxicity to photosynthetic pigments was not very significant (ANOVA, *P* > 0.05). It was observed that 100 *μ*M chromium treatment for 30 days reduced total chlorophyll, chlorophyll a, and chlorophyll b contents only by 8.52, 5.39, and 10.56% in* rosea* variety and 5.08, 5.65, and 4.72% in* alba* variety, respectively. Contrary to the results of chlorophyll, carotenoids content increases with the increasing concentration of Cr in the growth medium (Figure 1(b)). 54.5% increase was observed in the carotenoids content of* rosea* variety at 100 *μ*M Cr level which is quite significant (ANOVA *P* < 0.05), but in* alba* variety not much significant increase was noticed at any level of Cr treatment (Figure 1(b)).

### 3.2. Protein, Cysteine, Proline, and Nonprotein Thiol (NP-SH) Contents

The protein contents of the chromium treated plants were also found affected (ANOVA, *P* < 0.05) by chromium level in the soil (Figure 1(c)). A maximum reduction of 24.43% in protein content was observed at 100 *μ*M concentration in* alba* variety of* C. roseus, *whereas in* rosea* variety 22.95% reduction was found in protein content at 100 *μ*M level.

Chromium significantly (ANOVA, *P* < 0.05) increased the cysteine, proline, and nonprotein thiol (NP-SH) content in* C. roseus *leaves (Figures 1(d), 1(e) and 1(f)). The maximum increase in cysteine (46.32%) was found at 100 *μ*M level in* alba* variety, whereas maximum increase in proline (46.26%) and NP-SH (22.58%) was recorded at 50 *μ*M level in* alba* variety. In the* rosea* variety cysteine increased up to 20.83% at 50 *μ*M Cr level (Figure 1(d)), whereas proline and NP-SH increased up to 29.37% and 16.94% at 50 and 10 *μ*M level of Cr, respectively (ANOVA, *P* < 0.05) (Figures 1(e) and 1(f)).

### 3.3. Lipid Peroxidation and Membrane Permeability

**Figure 2:**
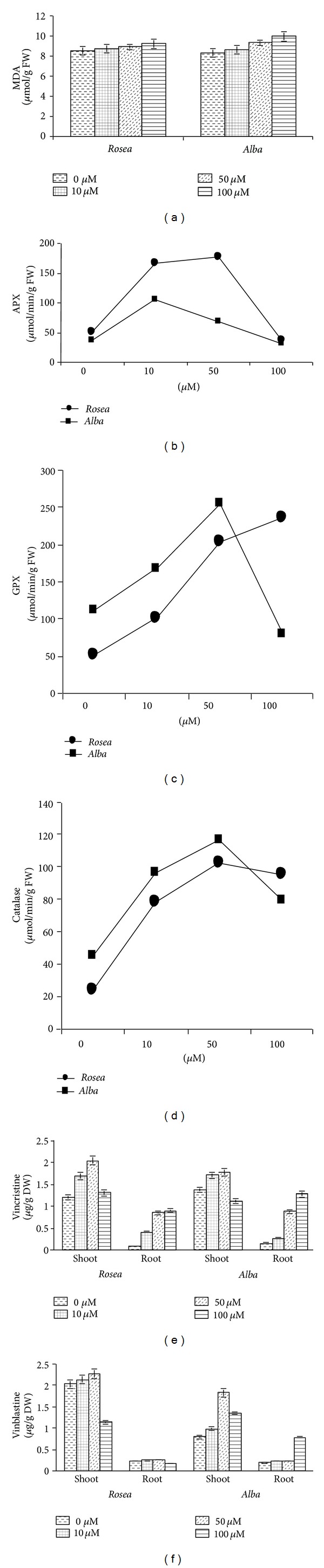
Effect of different chromium concentration on (a) MDA, (b) APX, (c) GPX, (d) catalase, (e) vincristine, and (f) vinblastine content of* C. roseus* after 30 days of Cr treatment. Mean ± SD (*n* = 3). ANOVA, *P* < 0.05.

During present study, a significant (ANOVA, *P* < 0.05) increase in malondialdehyde (MDA) content of* C. roseus *leaves was observed (Figure 2(a)) in the* alba* variety at 100 *μ*M chromium (after 30 days) in growth medium. It was noted that a maximum 19.53% increase of MDA content was found in the* alba* variety at 100 *μ*M level.

### 3.4. Antioxidant Enzymes

Heavy metal-induced reactive oxygen species in plants are quenched by a number of antioxidant enzymes. During the present study, it was observed that chromium toxicity resulted in a significant (ANOVA, *P* < 0.05) hyperactivity of ascorbate peroxidase (APX), guaiacol peroxidase (GPX) and catalase (CAT) enzymes in* C. roseus *leaves (Figures 2(b), 2(c), and 2(d)). The maximum stimulation of APX activity (3.49-fold) was observed when* rosea* variety plants were exposed to 50 *μ*M Cr and minimum at 100 *μ*M chromium exposure in* alba* variety (Figure 2(b)). However, peak stimulation of guaiacol peroxidase (GPX) (4.62 fold) was observed when plants were exposed to 100 *μ*M chromium in* rosea* variety for 30 days (ANOVA, *P* < 0.05) and minimum activity of GPX was found at 100 *μ*M chromium in* alba* variety (Figure 2(c)). Chromium significantly (ANOVA, *P* < 0.05) increased the catalase activity at 50 *μ*M Cr level in both varieties (Figure 2(d)). In the* rosea* variety 4.25 fold increase and in* alba* variety only 2.57 fold increases were found in catalase activity at 50 *μ*M Cr level.

### 3.5. Vincristine and Vinblastine Content

**Figure 3:**
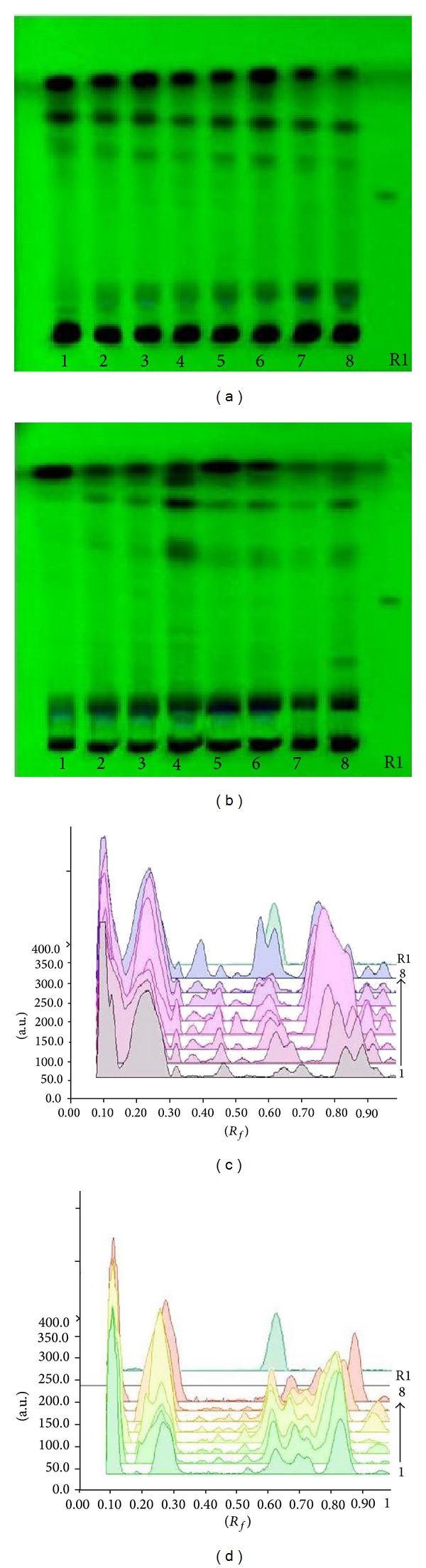
TLC fingerprint profile of methanolic extracts of control and treated plant along with marker compound of vincristine on HPTLC (E. Merck 60 F_254_) plate. 1–4:* C. roseus* variety* rosea*-control, 10, 50 and 100 *μ*M; 5–8:* C. roseus* variety* alba*-control, 10, 50 and 100 *μ*M; R1: vincristine marker. (a) Documentation of shoot samples under UV 254 nm; (b) documentation of root samples under UV 254 nm; (c) densitometric scan of shoot samples at 320 nm; (d) densitometric scan of root samples at 320 nm.

**Figure 4:**
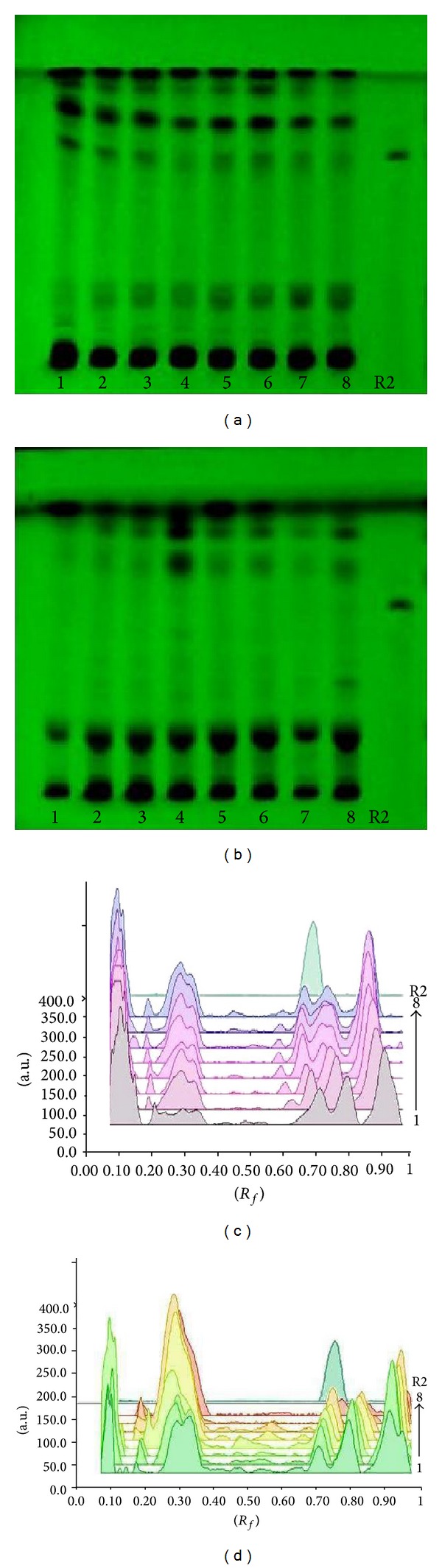
TLC fingerprint profile of methanolic extracts of control and treated plant along with marker compound of vinblastine on HPTLC (E. Merck 60 F_254_) plate. 1–4:* C. roseus* variety* rosea*-control, 10, 50 and 100 *μ*M; 5–8:* C. roseus* variety* alba*-control, 10, 50 and 100 *μ*M; R2: vinblastine marker. (a) Documentation of shoot samples under UV 254 nm; (b) documentation of root samples under UV 254 nm; (c) densitometric scan of shoot samples at 320 nm; (d) densitometric scan of root samples at 320 nm.

Chromium treatment to* C. roseus *affected vincristine and vinblastine content—active constituents of the plant (Student's *t*-test, *P* < 0.05). The chromium concentrations in the growth medium enhanced the vincristine content significantly up to 100 *μ*M Cr in the roots of both varieties, that is,* rosea* and* alba* (Figures 2(e) and 3), whereas in shoots it increases only up to 50 *μ*M Cr. 69.42% increase in vincristine content was observed in the shoots of* rosea* variety at 50 *μ*M chromium level (Figures 2(e) and 3). 2.29 fold increases in vinblastine content were found at the 50 *μ*M chromium level in the shoots of* alba* variety (Figures 2(f) and 4).

## 4. Discussion

*C. roseus *was able to tolerate 100 *μ*M chromium with some physiological and biochemical changes. This suggests this plant has a high adaptability to cope up with chromium stress. The roots accumulated more chromium than the leaves in all treatments.

Cr is a toxic, nonessential element to plants; hence, they do not possess specific mechanisms for its uptake. Therefore, the uptake of this heavy metal is through carriers used for the uptake of essential metals for plant metabolism [26]. The reason of the high accumulation in roots of the plants could be because Cr is immobilized in the vacuoles of the root cells, thus rendering it less toxic, which may be a natural toxicity response of the plant [27].

Chromium reduced the foliar contents of total chlorophyll, chl a, and chl b contents in* C. roseus*. This might be attributed to the toxicity of chromium to chlorophyll biosynthesis of the test plant through direct inhibition of photosynthesis [28]. In agreement with our results, the degeneration of chlorophyll is the most common response observed in plants exposed to elevated concentrations of various heavy metals [4, 5, 29–31]. Carotenoid, a nonenzymatic antioxidant, plays an important role in protection of chlorophyll pigment under stress conditions by quenching the photodynamic reactions and replacing peroxidation [32]. In agreement with our results, increased carotenoid content was also observed in heavy metal rich industrial effluent exposed* Capsicum annuum* [33]. An increase in carotenoid contents is considered as defense strategy of the plants to combat metal stress as observed in the present study.

Chromium has been reported to reduce foliar protein content in plants [31, 34]. In this case, also a small reduction in protein content was observed in chromium-treated* C. roseus*. As suggested by earlier workers [31, 34], protein degradation might be the result of increased activity of the protease or other catabolic enzymes, which were activated under chromium stress. It is also likely that chromium induced lipid peroxidation in* C. roseus *and fragmentation of proteins due to toxic effects of reactive oxygen species which led to reduced protein content [35].

Some nonenzymatic antioxidants like cysteine, nonprotein thiol, proline, and carotenoids may play a role in inducing resistance to metals by protecting labile macromolecules against attack by free radicals which are formed during various metabolic reactions leading to oxidative stress [36, 37]. During the present study, induced levels of NP-SH, carotenoids, and cysteine were observed in Cr-treated plants. A high level of NP-SH content might enable metabolites to function in free radicals and ROS detoxification, which are reductively detoxified by concomitant oxidation of sulfhydryl moieties to disulfides [38]. In the present study, the level of nonenzymatic antioxidants like cysteine, proline, and nonprotein thiol exhibited varied response to Cr depending on the metal concentration. Many environmental stresses have been reported to increase the level of proline in plants, such as heavy metals, UV radiation, temperature, and drought [39, 40]. During the present study, higher accumulation of proline in chromium-treated plants of* C. roseus *has been observed which might be attributed to the strategies adapted by plants to cope up with chromium toxicity as proline has multiple functions, such as, scavenger of free radicals, protector role of cytoplasmic enzymes, source of nitrogen and carbon for post stress growth, stabilizer of membranes, machinery for protein synthesis, and a sink for energy to regulate redox potential [40].

The chromium accumulation in* C. roseus *leads to various physiological and biochemical changes. Chromium in* C. roseus *promoted MDA production (a cytotoxic product of lipid peroxidation) through excessive generation of free radicals. Chromium-induced loss of membrane permeability coupled with increased MDA production has also been observed in* Vallisneria spiralis *[34] and* O. tenuiflorum *[5]. Many other authors have also reported an increase in MDA content under metal stress [29, 41, 42].

Chromium induced reactive oxygen species in plants [34, 43, 44]. Further, to mitigate and repair the damage initiated by reactive oxygen species, plants have evolved a complex system involving antioxidant enzymes. Ascorbate peroxidase (APX) is the member of the ascorbic acid-glutathione cycle and plays a crucial role in eliminating poisonous H_2_O_2_ from plant cells. In this study, two chromium concentrations, that is, 10 and 50 *μ*M induced APX activity in* C. roseus*, only at 100 *μ*M Cr level APX activity inhibited. This is in agreement with the results earlier described in different plants growing under heavy metal stress [45, 46]. Glutathione and free amino acids are known to induce heavy-metal tolerance by antioxidant action and metal chelating activity, respectively [47]. Recently, Zeng et al. reported that addition of glutathione (GSH) alleviated the reduction of plant growth and chlorophyll content but reduced malondialdehyde accumulation and increased the activities of the antioxidant enzymes in rice, suggesting that GSH may enhance antioxidant capacity in Cr-stressed plants [48]. Hence, it is possible that sulfate and iron supplementation can counter Cr toxicity in plants. It indicated that the activities of some antioxidant enzymes, including superoxide dismutase, catalase (CAT), and glutathione reductase, showed increase under Cr stress. Amongst various enzymes involved in quenching of reactive oxygen species, guaiacol peroxidase (GPX) and catalase have their importance in elimination of H_2_O_2_. The stimulated activities of these enzymes (GPX and catalase) found in this study led to the conclusion that elimination of H_2_O_2_ in* C. roseus* was achieved by APX, GPX, and catalase. Furthermore, GPX participates in the lignin biosynthesis and might build up a physical barrier against poisoning of the heavy metals. Therefore, hyperactivities of APX, GPX, and catalase in* C. roseus* might be attributed to the strategies adopted by the plant to overcome the toxicity of the chromium.

The secondary metabolites are formed under various stresses as a defense mechanism [2]. During the present investigation, chromium concentrations increased vincristine and vinblastine content in the treated plants. Vincristine and vinblastine are produced* in vivo *by the condensation of vindoline and catharanthine, both of which originate from the terpenoid indole alkaloid biosynthetic intermediate (+)-stemmadenine [49]. Vindoline, which is found only in the green parts of the plant, is biosynthesized from the branch-point intermediate tabersonine. This intermediate is formed from (+)-stemmadenine through the action of six enzymatic steps. These steps are sequentially catalysed by enzymes—T16H (tabersonine 16-hydroxylase), 16-hydroxytabersonine 16-O-methyltransferase (OMT), a hydroxylase (hydration), NMT (S-adenosyl-L-methionine: 16-methoxy-2,3-dihydro-3-hydroxy-tabersonine-N-methyltransferase), D4H (desacetoxyvindoline 4-hydroxylase), and acetyl-CoA: 4-O-deacetylvindoline 4-O-acetyl-transferase (DAT) [50]. Cacace et al. [51] identified Cr complex of flavonoid O-methyltransferase (OMT) and 16-hydroxytabersonine O-methyltransferase enzymes in cell cultures of* C. roseus*. It was noticed that flavonoid OMT does not synthesize flavonoids. Shukla et al. [49] reported 1-aminocyclopropane-1-carboxylate oxidase, which catalyses the final step in the biosynthesis of ethylene that is formed in response to wounding and stress, as well as during senescence. In the present study Cr stress may produce ethylene which stimulates accumulation of vincristine and vinblastine in* C. roseus*. Recently, it has also been reported that there was an improvement of indole alkaloid production in cell cultures of* C. roseus *treated by various chemicals, elicitors, earth elements and bioregulators [52–57]. In cell suspension culture, if the glucose concentration is increased in the medium, the secologanin got increased simultaneously with increase in the terpenoid indole alkaloid production in* Catharanthus* plants [58].

## 5. Conclusions

It is concluded from the present study that waste lands with chromium contamination may be used for the cultivation of* C. roseus*. However, extensive trials are required to find out a proper level of Cr in each type of soil. At higher concentration, the plants may be used for the extraction of alkaloids, but the care should be taken to consume plant parts as such because, in that case, level of Cr may be higher than permissible limit.

## Figures and Tables

**Table 1:** Chromium accumulation in *C. roseus*.

Cr. conc.	Cr accumulation (ppm)			
	*Rosea *		*Alba *	
	Shoot	Root	Shoot	Root
Control	n.d	n.d	n.d	n.d
10 *μ*M	2.79 ± 0.12	5.80 ± 0.15	2.78 ± 0.13	3.06 ± 0.15
50 *μ*M	10.36 ± 0.43	15.67 ± 0.56	15.03 ± 0.45	31.4 ± 0.98
100 *μ*M	25.96 ± 0.85	41.4 ± 1.05	25.28 ± 0.64	35.3 ± 0.95
